# Plasma levels of surfactant protein D and KL-6 for evaluation of lung injury in critically ill mechanically ventilated patients

**DOI:** 10.1186/1471-2466-10-6

**Published:** 2010-02-16

**Authors:** Rogier M Determann, Annick ANM Royakkers, Jack J Haitsma, Haibo Zhang, Arthur S Slutsky, V Marco Ranieri, Marcus J Schultz

**Affiliations:** 1 Department of Intensive Care Medicine, Academic Medical Center, Amsterdam, The Netherlands; 2 Department of Intensive Care Medicine, Tergooi Hospitals, Location Blaricum, Blaricum, The Netherlands; 3 Interdepartmental Division of Critical Care Medicine, St Michael's Hospital, University of Toronto, Canada; 4 Dipartimento di Anestesia e Rianimazione, Ospedale S Giovanni Battista-Molinette, Torino, Italy; 5 Laboratory of Experimental Intensive Care and Anesthesiology (LEICA), Academic Medical Center, Amsterdam, The Netherlands

## Abstract

**Background:**

Preventing ventilator-associated lung injury (VALI) has become pivotal in mechanical ventilation of patients with acute lung injury (ALI) or its more severe form, acute respiratory distress syndrome (ARDS). In the present study we investigated whether plasma levels of lung-specific biological markers can be used to evaluate lung injury in patients with ALI/ARDS and patients without lung injury at onset of mechanical ventilation.

**Methods:**

Plasma levels of surfactant protein D (SP-D), Clara Cell protein (CC16), KL-6 and soluble receptor for advanced glycation end-products (sRAGE) were measured in plasma samples obtained from 36 patients - 16 patients who were intubated and mechanically ventilated because of ALI/ARDS and 20 patients without lung injury at the onset of mechanical ventilation and during conduct of the study. Patients were ventilated with either a lung-protective strategy using lower tidal volumes *or *a potentially injurious strategy using conventional tidal volumes. Levels of biological markers were measured retrospectively at baseline and after 2 days of mechanical ventilation.

**Results:**

Plasma levels of CC16 and KL-6 were higher in ALI/ARDS patients at baseline as compared to patients without lung injury. SP-D and sRAGE levels were not significantly different between these patients. In ALI/ARDS patients, SP-D and KL-6 levels increased over time, which was attenuated by lung-protective mechanical ventilation using lower tidal volumes (*P *= 0.02 for both biological markers). In these patients, with either ventilation strategy no changes over time were observed for plasma levels of CC16 and sRAGE. In patients without lung injury, no changes of plasma levels of any of the measured biological markers were observed.

**Conclusion:**

Plasma levels of SP-D and KL-6 rise with potentially injurious ventilator settings, and thus may serve as biological markers of VALI in patients with ALI/ARDS.

## Background

Acute lung injury (ALI) and its more severe form, acute respiratory distress syndrome (ARDS) are life-threatening conditions with mortality rates up to 40% [1,2]. Prevention of additional lung injury caused by mechanical ventilation is a key element of care in these patients [3]. Clinical trials have demonstrated that non-protective forms of mechanical ventilation can cause additional pulmonary damage in patients with ALI/ARDS, frequently referred to as ventilator-associated lung injury (VALI) [2,4-6].

Development of VALI is not easily recognizable and therefore also not always preventable. One strategy frequently used to assess development of VALI is measurement of bronchoalveolar lavage fluid levels of proteins involved in the pathophysiology of lung injury. Several proteins have been suggested as possible biological markers of ALI/ARDS [7,8], and could potentially be used to monitor increased pulmonary inflammation associated with VALI [9]. However, obtaining bronchoalveolar lavage fluid may not always be possible and requires bronchoscopy expertise. In addition, respiratory and hemodynamic complications associated with bronchoscopy and subsequent lavage may hamper routine use of local levels of biological markers.

Systemic markers are far more easy to obtain, and can also be obtained more frequently. Several studies have shown systemic levels of proteins which occur naturally only in high levels in the pulmonary compartment to increase in patients with ALI/ARDS [10-13]. These include surfactant protein (SP)-D, Clara cell protein (CC16), KL-6 and soluble receptor for advanced glycation end-products (sRAGE). Although the process of entrance of proteins from the pulmonary compartment to the circulation has not yet been unraveled in detail, the increases in the systemic compartment have been ascribed to leakage across the alveolocapillary membrane, in association with the development of VALI [14]. ARDS is characterized by endothelial injury and increased vascular permeability [15] and it has been postulated that the alveolocapillary membrane has been damaged to such extent that pulmonary proteins diffuse under a concentration gradient to the circulation in patients with ALI/ARDS [16]. Increases in systemic levels of these proteins may thus be indicative of lung injury. Because of this pathophysiological relation, in theory these proteins may be better markers of ALI/ARDS as compared to proteins involved in inflammation as cytokines or clotting proteins, as they may be more specific for lung injury.

In the present study we investigated whether plasma levels of SP-D, CC16, KL-6 and sRAGE can be used in the evaluation of lung injury. We first analyzed whether plasma levels are associated with the lung injury score (LIS) [17] in patients with and without ALI/ARDS. We hypothesized plasma protein levels to be increased more in patients with the higher LIS. Furthermore, because worsening lung injury may be associated with bigger gaps in the alveolocapillary membrane, we hypothesized that the larger proteins SP-D and KL-6 would be more informative about the pulmonary status than levels of the smaller proteins CC16 and sRAGE. Second, we analyzed whether plasma levels of these biological markers change in patients ventilated with lung-protective or potentially injurious ventilation strategies. Third, the association between plasma levels and mortality, number of days on the ventilator and length of stay in hospital were studied.

## Methods

### Patients

Saved samples of patients with ALI/ARDS who participated in a previously reported multicenter study comparing lung-protective with conventional mechanical ventilation were collected [4]. Patients fulfilled the consensus criteria for ALI/ARDS and had an anticipated duration of mechanical ventilation of at least 48 hours. Next, samples of the first 20 consecutive patients without lung injury at onset of mechanical ventilation from a study comparing lung-protective with conventional mechanical ventilation were collected [18]. These patients also had an expected duration of mechanical ventilation of at least 48 hours. The study protocols of both studies were approved by the institutional review board of the participating hospitals. Informed consent was obtained from every patient or the closest relative.

### Mechanical ventilation protocols

ALI/ARDS patients were randomized to one of two mechanical ventilation protocols as described previously [4]. In short, in the conventional protocol the respiratory rate was set at 10 - 15 breaths per minute and the tidal volume was set to maintain an arterial carbon dioxide pressure of 35 - 40 mm Hg. However, for safety reasons, the plateau pressure could not exceed 35 cm H_2_O irrespective of arterial carbon dioxide pressure. In the lung-protective protocol, the plateau pressure was set at the upper inflection point of the pressure volume curve of the lung while the level of positive end expiratory pressure (PEEP) was set 1 - 3 cm H_2_O above the lower inflection point.

Patients without lung injury at onset of mechanical ventilation were randomized to a conventional protocol using tidal volumes of 10 ml/kg ideal body weight (IBW), or a lung-protective protocol using tidal volumes of 6 ml/kg IBW. Respiratory rate and levels of PEEP were set at the discretion of the attending physician.

### Sample collection

Blood samples were drawn from a central venous line into vacutainers containing heparin on the day of inclusion and after 48 hours. Immediately thereafter, samples were centrifuged at 1500 × g for 10 minutes. The supernatant was collected and samples were stored at - 80°C until analysis.

### Measurements

Levels of SP-D, CC16 and sRAGE were measured with an enzyme-linked immunoassay (ELISA) as described before [19]. Different from previous measurements another capture antibody was used (clone HM3022, HyCult, Uden, The Netherlands). An enzyme immuno assay for measurement of KL-6 was kindly offered by Sanko Junyaku Co Ltd (Tokyo, Japan).

### Lung injury score

To assess lung injury in patients the lung injury score (LIS) was determined [17]. This was done by two members of the study group who were blinded for the measurements of pulmonary proteins.

### Statistical analysis

Descriptive results of continuous data were expressed as means with standard deviations for parametric data or as medians with ranges for non-parametric data. To identify differences between groups analysis of variance, the Kruskal-Wallis, χ^2^- or Fisher's exact statistics were used as appropriate. Baseline correlations between protein levels and LIS were assessed with Spearman's rho. To study the relation between pulmonary protein levels and the LIS over time, a linear mixed model was constructed. In this model serial measurements were combined to investigate the association between LIS and protein levels. In this way, we were able to investigate the effect of an increase in protein level on the LIS and thereby investigating our hypothesis that larger proteins would be more informative on changes in LIS. We repeated this analysis while controlling for presence of pulmonary disease or non-pulmonary disease and age. To investigate whether mechanical ventilation protocol would influence protein levels, a second linear mixed model was constructed in which changes in protein levels over time were studied while controlling for mechanical ventilation protocol (conventional or protective). Finally, the association between protein levels and mortality, length of stay in hospital and number of days on the ventilator were studied. A two-sided P-value < 0.05 was considered as statistically significant. All analyses were done with SPSS, version 14.

## Results

### Patients

Sixteen patients with ALI/ARDS and 20 patients without lung injury at the onset of mechanical ventilation were studied. The demographic data and baseline data are presented in table 1. There were no differences in baseline characteristics, neither between the ALI/ARDS groups nor in between the patients groups without lung injury at onset. Patients in the second study did not develop ALI/ARDS during conduct of the study. As expected, LIS was higher in patients with ALI/ARDS as compared to patients without lung injury.

**Table 1:** Baseline characteristics

	ALI/ARDS Lung-protective (n = 8)	ALI/ARDS Conventional (n = 8)	P-value	no lung injury Lung-protective (n = 10)	no lung injury Conventional (n = 10)	P-value
Age (yrs)	58 ± 18	58 ± 22	0.97	57 ± 19	58 ± 19	0.82
Male sex, No. (%)	4 (50%)	4 (50%)	0.80	7 (70%)	7 (70%)	1.00
APACHE II score	14.8 ± 2.4	14.1 ± 2.0	0.58	13.8 ± 2.3	15.2 ± 1.5	0.78
LIS	2.2 ± 0.4	2.3 ± 0.5	0.74	0.7 ± 0.7	0.7 ± 0.7	0.92
Admission diagnosis			0.43			0.41
Resuscitation				7	4	
Neurological disease				2	2	
Cardiopulmonary surgery					2	
Sepsis	4	3			1	
Trauma	1	3		1	1	
Pneumonia	3	2				

ALI acute lung injury; ARDS acute respiratory distress syndrome; APACHE acute physiology and chronic health evaluation; LIS lung injury score. Values are displayed as means with standard deviation.

### Respiratory parameters

**Figure 1:**
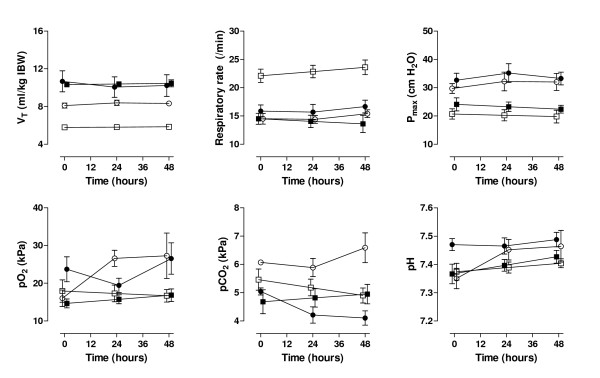
**Mechanical ventilation parameters**. Mechanical ventilation parameters in patients with ALI/ARDS (circles) and without lung injury at onset of mechanical ventilation (squares). Open symbols: patients ventilated with a lung-protective mechanical ventilation strategy with lower tidal volumes; closed symbols: patients ventilated with a potentially lung-injurious strategy using conventional tidal volumes. V_T_, tidal volume; P_max_, maximum airway pressure.

Tidal volumes differed significantly between the two ventilation strategies, both in ALI/ARDS patients and patients without lung injury at onset of mechanical ventilation (figure 1). While maximum airway pressures were significantly higher in both ALI/ARDS groups, tidal volumes and respiratory rates applied in patients without lung injury at onset of mechanical ventilation were comparable with those in patients in the lung-protective ALI/ARDS group.

### Association between proteins levels and LIS

**Figure 2:**
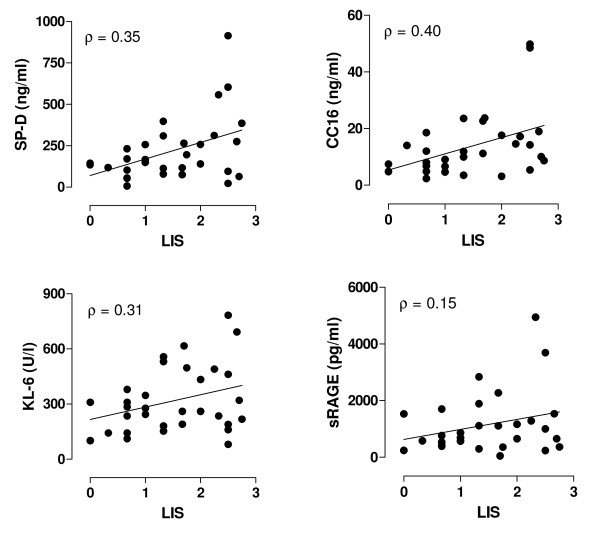
**Baseline biomarker levels and lung injury score**. Scatter plots of baseline levels of surfactant protein D (SP-D), Clara cell protein (CC16), KL-6 and soluble receptor for advanced glycation end products (sRAGE) as a function of lung injury score (LIS). The correlation is expressed as Spearman's rho (ρ).

The baseline protein levels are displayed in table 2. ALI/ARDS patients had higher plasma levels of SP-D, CC16 and KL-6, as compared to patients without lung injury at onset of mechanical ventilation, although statistical significance was not reached for SP-D. Plasma levels of sRAGE were comparable between patients from the two studies. Baseline correlations between LIS and protein levels were significant for SP-D, CC16 and KL-6, (ρ = 0.35, 0.40, and ρ = 0.31, respectively, *P *< 0.01 for all, figure 2).

**Table 2:** Baseline plasma protein levels

	ALI/ARDS (n = 16)	No lung injury (n = 20)	P-value
SP-D (ng/ml)	275 [80 - 462]	140 [84 - 216]	0.09
CC16 (ng/ml)	14.3 [9.0 - 19.0]	7.7 [4.8 - 13.5]	0.03
KL-6 (U/l)	477 [287 - 636]	240 [160 - 304]	0.001
sRAGE (pg/ml)	1000 [300 - 1500]	725 [532 - 1660]	0.59

ALI acute lung injury; ARDS acute respiratory distress syndrome; SP-D surfactant protein D, CC16 Clara cell protein, sRAGE soluble receptor for advanced glycation end products. Values are displayed as medians with interquartile range.

Linear mixed model analysis showed that both CC16 and KL-6 were associated with the LIS over time but the strongest association was found with CC16. A change of 10 ng/ml of CC16 was associated with a change of 0.4 (95%-CI 0.2 - 0.6) of LIS (*P *= 0.001). A change of 100 U/l of KL-6 was associated with a change of 0.2 (95%-CI 0.08 - 0.28) of LIS (*P *= 0.001). On multivariate analysis, controlling for age and presence of pneumonia, KL-6 was an independent predictor of the LIS (*P *= 0.004) together with presence of pneumonia (*P *= 0.03). An increase of 100 U/l of KL-6 was associated with an increase of 0.18 (95%-CI 0.07 - 0.30) of LIS while presence of pneumonia was associated with an increase of 0.97 (95%-CI 0.8 - 1.15).

### Plasma levels of pulmonary proteins with different ventilator settings

In the next step we investigated the relation between mechanical ventilation protocol and plasma protein levels. As mechanical ventilation protocols were different amongst patients with and without ALI/ARDS, this analysis was performed for each group separately.

**Figure 3:**
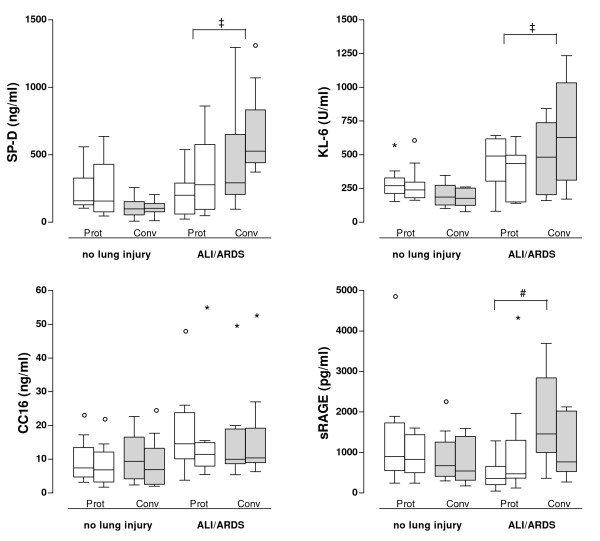
**Biomarker levels per group at two time points**. Levels of surfactant protein D (SP-D), Clara cell protein (CC16), KL-6 and soluble receptor for advanced glycation end products (sRAGE) in patients without lung injury at onset of mechanical ventilation (no lung injury) and ALI/ARDS patients (ALI/ARDS), ventilated with either lung-protective mechanical ventilation (open boxes) or potentially lung-injurious mechanical ventilation (grey boxes). In each group, the left bar represents T = 0 hours, the right bar represents T = 48 hours after inclusion. Box plots represent 25^th^, 50^th ^and 75^th ^percentile; whiskers represent values within 10^th ^and 90^th ^percentile; "°" represent outliers and "*" represent extreme outliers. # *P *< 0.05 for difference in absolute values between groups at baseline; ‡ *P *< 0.05 for difference in increases over time between groups.

In univariate analysis, plasma levels of SP-D increased significantly after 48 hours in ALI/ARDS patients (275 [80-462] ng/ml to 487 [278-776] ng/ml, *P *< 0.001). This increase was significantly smaller in ALI/ARDS patients ventilated with the lung-protective strategy (*P *= 0.02 versus conventional). Levels of CC16 remained unchanged over time in both ALI/ARDS groups and no significant differences were seen between these groups (*P *= 0.27 versus conventional). Levels of KL-6 increased in ALI/ARDS patients who received conventional mechanical ventilation but remained stable in the lung-protective ALI/ARDS group (figure 3, *P *= 0.02 versus conventional). In contrast, sRAGE levels declined in the conventional group while they remained constant in the lung-protective group (*P *= 0.03 versus lung-protective). In patients without lung injury at onset of mechanical ventilation no changes were seen over time in any of the studied markers.

We performed a multivariate analysis controlling for presence of pneumonia only for SP-D and KL-6. In multivariate analysis, both SP-D and KL-6 levels were significantly influenced by mechanical ventilation protocol (*P *= 0.04) while presence of pneumonia was not significantly associated.

### Association between plasma protein levels and clinical outcome

We examined whether plasma levels of biomarkers were related to mortality, length of stay (LOS) and the number of days on the ventilator in the ALI/ARDS group. Five ALI/ARDS patients of the conventional group and 3 ALI/ARDS patients of the lung-protective group died. Non-survivors were more likely to show increases in SP-D and KL-6 levels as compared to survivors (*P *= 0.01). No association was found between CC16 or sRAGE levels and mortality. Both the baseline level of SP-D and KL-6 were significantly associated with the number of days on the ventilator (*P *= 0.04 for both) and length of stay in hospital (*P *= 0.04 and *P *= 0.03, respectively)

## Discussion

We investigated whether plasma levels of SP-D, CC16, KL-6 and sRAGE could be used for evaluation of lung injury in critically ill mechanically ventilated patients. Plasma levels of both CC16 and KL-6 were associated with the LIS but the strongest association was found with CC16. However, while CC16 levels remained unchanged over time, SP-D and KL-6 levels increased in ALI/ARDS patients when ventilated with a conventional mechanical ventilation protocol. Moreover, plasma levels of SP-D and KL-6 were significantly associated with mortality, the number of days on the ventilator and length of stay in hospital in patients with ALI/ARDS.

Several limitations of our study have to be acknowledged. First, we included a limited number of patients. The primary goal of the study was to investigate whether biological markers were associated with the LIS in mechanically ventilated patients. As a second goal we investigated whether mechanical ventilation strategy would influence levels of these biological markers. Our sample size was limited because it is unethical to subject ALI/ARDS patients to a potentially injurious strategy for a prolonged period of time. As such, we used samples collected in the course of a previous clinical trial [4], before it was certain that lung-protective mechanical ventilation protects against VALI. A second shortcoming is that we included patients with different etiologies for ALI/ARDS. Since the pathophysiology of ALI/ARDS caused by indirect causes (e.g., sepsis) may be different from direct causes (e.g., pneumonia), it may be that plasma levels of pulmonary proteins are also different patients or pneumonia patients with ALI/ARDS. While we performed multivariate analysis controlling for this issue, this may still have influenced our results due to the small sample size. Also, a number of patients without lung injury at onset of mechanical ventilation had cardiopulmonary resuscitation after cardiac arrest or had severe trauma which may have caused slight contusion of the lungs which may have influenced baseline plasma levels of pulmonary proteins. Finally, we studied patients ventilated with a ventilation protocol which is no current practice anymore. However, in order to study the possibility that biomarkers may detect VALI, a setting is needed in which patients have obvious VALI. From there, additional studies need to investigate whether these biomarkers can detect beginning lung injury in patients ventilated with current ventilation strategies. It has been shown that protective mechanical ventilation strategies may still be injurious in some patients with severe lung injury [9]. Although CC16 correlated better with the LIS than SP-D or KL-6, our results showed that the latter two biomarkers increase in patients with VALI. Therefore, future studies should investigate whether these biomarkers are able to detect lung injury in patients ventilated with current ventilation strategies.

Our results show that biological markers may be useful in monitoring lung injury in critically ill mechanically ventilated patients. While in univariate analysis, SP-D, CC16 and KL-6 were all associated with the LIS, in multivariate analysis only KL-6 was independently associated with the LIS. Furthermore, our data suggest that plasma KL-6 levels might be used to monitor lung injury in patients receiving mechanical ventilation. However, before biomarkers can be implemented as a surrogate method to diagnose lung injury, a large clinical study is needed to answer the question whether biomarkers or a combination of biomarkers with clinical parameters are better than the LIS or NAECC criteria. In this light, it is also of interest to test biomarker levels against methods that measure extravascular lung water or radionuclide methods of assessing capillary protein permeability [20]. Although these methods are more time-consuming than biomarker measurement, no study has ever compared these methods with these biomarkers in detecting lung injury.

We did not observe any differences in the non-ALI group between conventional and lower tidal volume strategies. The small number of patients and the heterogeneity of this patients group may have accounted for this negative finding. Previous studies suggest that lung injury develops in non-ALI patients if submitted to conventional tidal volumes during surgery [21,22]. These studies were however much more controlled, i.e. sampling time and more comparable patient groups, which may have lead to lower variability in the measured variables.

Our data are, at least in part, in line with results from a previous study. In a sub-analysis of the ARDS Network trial comparing lower tidal volumes with conventional tidal volumes systemic levels of SP-D increased during the first 3 days of mechanical ventilation [10]. We found both SP-D and KL-6 to increase in patients who were ventilated with a conventional ventilation strategy. Two pathophysiological processes may account for this finding. First, in patients with ALI/ARDS the lung blood barrier is damaged which may result in increased passive leakage of SP-D and KL-6 from the lungs to the circulation. Secondly, ALI/ARDS is also characterized by strong proliferation of alveolar type II cells. Indeed, experimental rat models showed systemic SP-D levels to increase after instillation of keratinocyte growth factor in the lungs which causes strong proliferation of alveolar type II cells without signs of lung injury [23]. Moreover, the production of SP-D and KL-6 by alveolar type II cells in humans is increased during states of proliferation [24]. Therefore, both damage to the alveolocapillary membrane and proliferation of alveolar type II cells may result in increased plasma levels of SP-D and KL-6.

Surprisingly, we did not show any increases in CC16 or sRAGE levels in ALI/ARDS patients ventilated with conventional mechanical ventilation. Both SP-D and KL-6 are produced by alveolar type II cells during the proliferative phase while RAGE and CC16 are not. Elevated levels of CC16 or sRAGE during ALI/ARDS have been ascribed to damaged epithelium (type I cells) with an ensuing increased alveolar epithelial permeability [11]. Although proliferation is likely to occur later in the sequence of ALI/ARDS, the absence of increased CC16 or sRAGE levels in the conventional group may indicate proliferation of alveolar type II cells as the primary source of increased SP-D and KL-6 levels.

As explained above, a problem with using serum SP-D and KL-6 as biological markers is that we don't fully understand the biology leading to the increased systemic levels of these proteins in patients with ALI/ARDS. From studies in patients with interstitial lung disease, which is characterized by severe proliferation and fibrosis, it is known that increases in plasma levels of SP-D and KL-6 imply enhanced proliferation and worsening of the disease [25]. Therefore, increases in plasma levels of SP-D and KL-6 in patients with ALI/ARDS may indicate progression to the proliferative phase. As with patients with interstitial fibrosis, patients with the most abundant proliferation may have the worst outcomes. We observed the largest increases in SP-D and KL-6 levels in patients with the worst outcomes. However, these markers were measured in the periods of 1-3 days after diagnosis was made. It was therefore unknown whether extensive proliferation was already present. If these markers are to be implemented in clinical practice, more knowledge on these pathophysiological issues in ALI/ARDS patients is needed.

## Conclusions

Systemic levels of lung proteins may be useful for evaluation of lung injury in critically ill mechanically ventilated patients. Both systemic SP-D and KL-6 levels increase in ALI/ARDS patients who are ventilated with potentially injurious forms of mechanical ventilation. Moreover, systemic levels of SP-D and KL-6 are associated with mortality, duration of mechanical ventilation and length of stay in hospital. Before these markers can by implemented in routine clinical practice, not only additional information on alveolar type II cells and SP-D and KL-6 production in ALI/ARDS patients is needed but also a study comparing the use of biomarkers with current clinical ALI/ARDS scores as the LIS and NAECC criteria.

## Competing interests

The authors declare that they have no competing interests.

## Authors' contributions

Conception and design were done by RMD and MJS. The acquisition and assembly of data was done by RMD, AANMR, JH, HZ, VMR and ASS. Analysis and interpretation of the data was done by RMD and MJS. The first draft of the manuscript was made by RMD and MJS. Critical revision of the manuscript for important intellectual content was done by RMD, AANMR, JH, HZ, VMR, ASS and MJS. All authors read and approved the final version of the manuscript.

## Pre-publication history

The pre-publication history for this paper can be accessed here:

http://www.biomedcentral.com/1471-2466/10/6/prepub
